# Diagnosis of acute appendicitis at a pediatric emergency department within a general hospital

**DOI:** 10.1007/s10140-026-02442-w

**Published:** 2026-02-24

**Authors:** Cade A. Johnson, Catherine M. Pivalizza, Hei Kit Chan, Hannah Smith, Megan K. Long, Robert Lapus, KuoJen Tsao, Susan John, Irma T. Ugalde

**Affiliations:** 1 Department of Emergency Medicine, McGovern Medical School at The University of Texas Health Science Center, Houston, TX USA; 2 McGovern Medical School at The University of Texas Health Science Center, Houston, TX USA; 3 University of Texas Health Science Center at Houston School of Public Health, Houston, TX USA; 4 Department of Pediatric Emergency Medicine, Tristar Emergency Medicine Residency Training Program, Nashville, TN USA; 5 Memorial Hermann Hospital, Sugarland, TX USA; 6 Department of Pediatric Surgery and Neurosurgery, McGovern Medical School at The University of Texas Health Science Center, Houston, TX USA; 7 Department of Diagnostic and Interventional Radiology, McGovern Medical School at The University of Texas Health Science Center, Houston, TX USA; 8 Department of Pediatrics, Section of Emergency Medicine, University of Chicago Pritzker School of Medicine, Chicago, IL USA

**Keywords:** Acute appendicitis, Ultrasound (US), Magnetic resonance imaging (MRI), Pediatric Emergency Department (ED), Diagnostic accuracy

## Abstract

**Purpose:**

Ultrasound (US) has evolved as the principal imaging modality in the evaluation of pediatric appendicitis due to inherent advantages but may vary in performance based on setting. Rapid focused Magnetic Resonance Imaging (rapid MRI) is a feasible alternative, but emergency department (ED) length of stay (LOS) may be impacted. We wished to determine the performance and LOS of US and rapid MRI in a pediatric ED within a general hospital in the evaluation of appendicitis.

**Methods:**

We conducted a retrospective diagnostic accuracy study of patients < 18 years of age with abdominal pain receiving imaging for evaluation of appendicitis (US or rapid MRI). We report descriptive statistics on demographics and clinical characteristics of the study population. We conducted a sensitivity analysis by the different imaging type and combinations performed.

**Results:**

Sensitivity and specificity for US and rapid MRI were 65.3% (95% CI: 57.5%–72.5%); 97.0% (95% CI: 94.5%–98.5%) and 98.3% (95% CI: 90.6%–100.0%); 96.1%, (95% CI: 91.1%–98.7%), respectively. US alone was associated with the shortest median ED LOS (5.3 hours, IQR 3.9–7.8), followed by rapid MRI alone (7.9 hours, IQR 5.4–8.6). US with subsequent rapid MRI was associated with the longest ED LOS (10.6 hours, IQR 7.6–13.3).

**Conclusion:**

US was nondiagnostic for a substantial number of studies performed resulting in suboptimal sensitivity for the diagnosis of appendicitis. Rapid MRI had better sensitivity but a greater LOS. Future work should focus on determining potential barriers that exist and incorporating strategies to enhance US performance and/or decrease rapid MRI LOS.

**Graphical Abstract:**

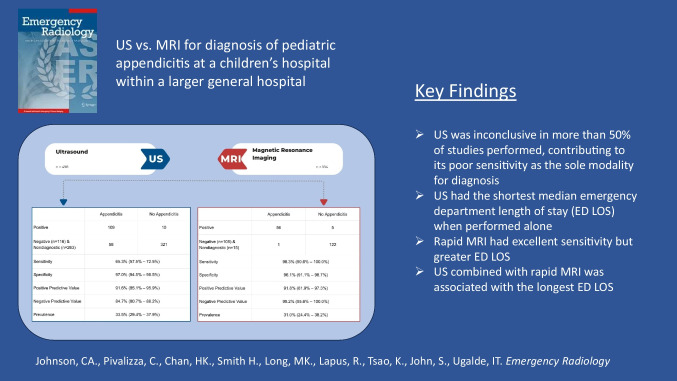

**Supplementary Information:**

The online version contains supplementary material available at 10.1007/s10140-026-02442-w.

## Introduction

Appendicitis poses a significant pediatric health challenge, with an incidence of 0.5–1.5% worldwide, making it one of the primary surgical emergencies in children [1]. An accurate, prompt diagnosis is critical and is achieved through a comprehensive approach that includes clinical assessment, laboratory work, and imaging modalities to prevent serious complications such as appendiceal rupture, peritonitis, and intraabdominal abscess formation [2, 3].

Ultrasound (US) and Computed Tomography (CT) have long been recognized as key imaging modalities in the evaluation of pediatric appendicitis, each with advantages and potential limitations [4]. US is the recommended first-line modality by the American College of Radiology [5]. It is non-invasive, cost-effective, and avoids radiation exposure, although it can be limited by body habitus and overlying bowel gas [6]. US is operator-dependent, and not all facilities have the resources and expertise to use it as the first-line modality in pediatric patients. Despite the potential risk of ionizing radiation, CT has been used regularly for pediatric patients because it is widely available and accurate [7, 8]. Non-contrast rapid acquisition Magnetic Resonance Imaging (rapid MRI) is an alternative to CT [9–12]. Rapid MRI provides non-ionizing imaging with high sensitivity (96–97%) and specificity (96–97%) [9, 13]. The clinical setting can impact diagnostic accuracy and approach: community hospitals have previously been associated with 4.4-fold higher odds of obtaining pre-operative CT scans, rather than US and rapid MRI [14, 15].

The primary objective of this study was to compare the diagnostic accuracy of US and rapid MRI for pediatric appendicitis and analyze emergency department (ED) length of stay (LOS) within a pediatric ER nestled in a larger general urban hospital. We hypothesized that rapid MRI would offer greater accuracy, though potentially at the expense of efficiency.

## Methods

### Study design/setting

We conducted a retrospective diagnostic accuracy study at Children’s Memorial Hermann Hospital (CMHH) between 01/01/2019 and 06/30/2019. This study was approved by our institutional review board under expedited review (IRB protocol: HSC-MS-19–1049). The pediatric emergency department (ED) is located within CMHH in the Texas Medical Center and acts as the primary catchment area for the larger hospital network of 11 Memorial Hermann Hospitals (MHH) in the greater Houston, Texas area. The pediatric ED at CMHH has approximately 20,000 emergency visits annually, and the Children’s Hospital performs over 6,400 pediatric surgeries. Each MHH ED has 24/7 access to US, rapid MRI, and CT. Experienced sonographers perform US studies, and all imaging studies are interpreted by attending radiology physicians. Residents provide preliminary interpretations between 5 p.m. and 8 am, with final pediatric radiology attending reports available after 8 am. In the case of repeated images within the same modality, the most recent image was selected for analysis.

### Study population & protocol

Patients less than 18 years of age with abdominal pain and a differential diagnosis including appendicitis, as determined by the care team in the ED, who received imaging to rule out appendicitis (US, rapid MRI, CT) at CMHH or outlying MHH hospitals were included in the study in a consecutive series. Choice of imaging study was determined by the treating clinician team. The ED protocol at CMHH encouraged the use of US first before the use of MRI and generally discouraged the use of CT. Patients were excluded from the study if they were transferred to CMHH without legible or available imaging reports.

### Measurements & outcomes

Electronic records of eligible patients were reviewed by study team members and then adjudicated by an additional blinded team member if there were discrepancies. For both US and rapid MRI reports, right lower quadrant (RLQ) US and rapid abdominal/pelvic MRI without contrast templates, respectively, were employed (appendix a, appendix b). Rapid MRI protocol typically lasted 20–30 minutes occasionally requiring midazolam (Versed) for anxiolysis, but not sedation (appendix c). RLQ US images were reviewed for: visualization of the appendix (partial or full), appendix size, free and contained fluid, compressibility, appendicolith, hyperemia, fat edema, bowel peristalsis, and localized tenderness over the appendix. Rapid MRI images were reviewed for: visualization of the appendix, size and location, free fluid (including trace or physiologic), periappendiceal edema, restricted diffusion, and appendicolith. Chronological order of imaging allowed for determination of the last image used for affirmative diagnosis in patients with appendicitis. The most up-to-date imaging study was used for analysis for patients who underwent repeat imaging of a specific modality. Patients without appendicitis were considered true negatives for all modalities unless chart review indicated that the patient returned to the ED for appendicitis during the study period. Imaging results were categorized as diagnostic or non-diagnostic. Diagnostic findings had clear indicators as described above, confirming or excluding a diagnosis of appendicitis. A nondiagnostic study had equivocal findings which could not confirm or exclude appendicitis. In patients who underwent surgery, appendicitis was confirmed by a positive surgical pathology report. Results were classified as simple or complicated (gangrenous, perforated).

Length of stay (LOS) was derived from the patient chart as the time between ED registration and departure, including either admission for surgery or discharge home. For patients transferred from another ED in the MHH system, the LOS was calculated by combining the time from ED registration at the original ED to transfer and the LOS at CMHH.

### Data analysis

We displayed the distribution of each imaging combination and examined patient demographics and clinical characteristics using descriptive statistics stratified by diagnosis (diagnostic and nondiagnostic) and specific imaging combinations: a) Overall, b) US only, c) MRI only, d) CT only, e) US & MRI, f) US & CT, g) US & MRI & CT. Median and inter-quartile range were reported for continuous variables, while frequencies and percentage were reported for categorical variables.

Continuous variables were analyzed using the Wilcoxon rank-sum test for inference on difference between diagnosis results, while categorical variables were analyzed with Pearson’s 𝜒2 test or Fisher’s exact test (for variables with expected frequencies less than 5) for inference on association with diagnosis results. We performed sensitivity and specificity analysis for each imaging study (US, rapid MRI, and CT) for: 1. diagnostic imaging (positive or negative) and 2. overall imaging (diagnostic and nondiagnostic). Positive diagnostic imaging represented exams where the appendix was clearly identified and the diagnosis of appendicitis was made in the radiology report. Negative diagnostic imaging represented exams where the appendix was clearly identified and considered not to be appendicitis in the radiology report. Non-diagnostic tests were exams where the appendix may not have been identified and/or the diagnosis was not conclusive. These exams usually led to further imaging unless the patient’s exam was no longer suspicious for appendicitis and the clinical care team forewent any further imaging. An inconclusive study negatively impacted clinical practice and when found to be positive with additional imaging was classified as a false negative test in the second analysis (B) of sensitivity and specificity. If found to truly be negative with the addition of other imaging, it represented a true negative test.

For patients with confirmed appendicitis, we compared diagnostic to nondiagnostic imaging for simple versus complicated/perforated appendicitis through Pearson’s 𝜒2 test and Fisher’s exact test (for variables with expected frequencies less than 5). It was not feasible to perform sensitivity and specificity analysis on these two groups as only patients with appendicitis (positive disease) were included in this comparison and no patients without appendicitis (negative disease).

We followed the Standards for Reporting of Diagnostic Accuracy (STARD) guidelines.

## Results

Over the 6-month period, after exclusions, 587 out of the 610 eligible patients were included in the study. Patient demographics and clinical characteristics of the cohort are demonstrated in **Table** **1**. The median age of the cohort was 10 years, with a nearly equal distribution of male (306, 52.1%) and female (281, 47.9%) patients. Of the cohort, 236 (40%) patients were ultimately diagnosed with and treated for appendicitis. Patients with appendicitis were older, more frequently male, and there was a higher proportion of Hispanic and a lower proportion of Black patients. Patients with appendicitis had a higher frequency of all clinical characteristics evaluated except fever and ED LOS compared to patients without appendicitis.

**Table 1:** Patient demographics and clinical characteristics comparing patients with and without appendicitis

	Appendicitis N = 236	No Appendicitis N = 351	P-Value ^a^
*Age (years), median (IQR* ^*b*^*)*	10.6 (8.1–14.0)	9.0 (6.0–12.0)	< 0.01
*Sex, n (%)*			< 0.01
Female	88 (37.3)	193 (55.0)	
Male	148 (62.7)	158 (45.0)	
*Race & Ethnicity, n (%)*			< 0.01
White	62 (26.3)	98 (27.9)	
Black	22 (9.3)	55 (15.7)	
Asian	3 (1.3)	9 (2.6)	
Hispanic	30 (12.7)	0 (0.0)	
Other/Unknown	119 (50.4)	189 (53.8)	
*Fever, n (%)*			0.34
Yes	81 (34.3)	144 (41.0)	
No	138 (58.5)	207 (59.0)	
*Vomiting, n (%)*			< 0.01
Yes	177 (75.0)	227 (64.7)	
No	42 (17.8)	118 (33.6)	
*RLQ Tenderness, n (%)*			< 0.01
Yes	188 (79.7)	228 (65.0)	
No	31 (13.1)	114 (32.5)	
*WBC > 10k, n (%)*			< 0.01
Yes	180 (76.3)	120 (34.2)	
No	39 (16.5)	123 (35.0)	
*ED LOS (hours), median (IQR* ^*b*^*)*	7.2 (4.5–10.3)	6.2 (4.5–9.6)	0.14

a: P-values for age were derived from Wilcoxon rank sum test. P-value for sex was derived from Pearson’s 𝜒2 test, while P-value for race & ethnicity was derived from Fisher’s exact test.

b: IQR = inter-quartile range.

*ED LOS* emergency department length of stay

Patient clinical characteristics grouped by imaging modality are shown in Table 2. Patients who had combined US & CT or rapid MRI alone (median age 12.6, 11 yrs., respectively) were older than patients with US only or US & rapid MRI (median age 9.0 yrs). Patients with either US or rapid MRI alone, were more frequently male, while those with combined imaging were more frequently female. Ultrasound alone was associated with the shortest median ED LOS, followed by CT alone, and subsequently rapid MRI alone. US combined with rapid MRI was associated with the longest ED LOS. One patient had all three modalities performed with a total LOS of 5.9 hours.

**Table 2:** Patient clinical characteristics grouped by modalities performed

	Overall N = 587	US only N = 330	MRI only N = 28	CT only N = 61	US & MRI N = 155	US & CT N = 12	US & MRI & CT N = 1	P-Value ^a^
*Fever, n (%)*								0.77
Yes	225 (38.3)	125 (37.9)	8 (28.6)	22 (36.1)	65 (41.9)	5 (41.7)	0 (0.0)	
No	345 (58.8)	192 (58.2)	20 (71.4)	37 (60.7)	89 (57.4)	6 (50.0)	1 (100.0)	
*Vomiting, n (%)*								0.76
Yes	404 (68.8)	223 (67.6)	17 (60.7)	45 (73.8)	110 (71.0)	8 (66.7)	1 (100.0)	
No	160 (27.3)	90 (27.3)	11 (39.3)	14 (23.0)	42 (27.1)	3 (25.0)	0 (0.0)	
*RLQ Tenderness, n (%)*								0.15
Yes	416 (70.9)	221 (67.0)	21 (75.0)	50 (82.0)	116 (74.8)	7 (58.3)	1 (100.0)	
No	145 (24.7)	90 (27.3)	7 (25.0)	8 (13.1)	36 (23.2)	4 (33.3)	0 (0.0)	
*WBC > 10k, n (%)*								0.04
Yes	162 (27.6)	93 (28.2)	10 (35.7)	11 (18.0)	46 (29.7)	2 (16.7)	0 (0.0)	
No	300 (51.1)	144 (43.6)	17 (60.7)	48 (78.7)	81 (52.3)	9 (75.0)	1 (100.0)	
***ED LOS (hours),*** *median (IQR* ^*b*^*)*	6.5 (4.5–9.9)	5.3 (3.9–7.8)	7.9 (5.4–8.6)	6.1 (4.4–9.4)	10.6 (7.6–13.3)	9.2 (2.9–10.9)	5.9 (5.9–5.9)	< 0.01

a: P-value for ED length of stay was derived from Kruskal-Wallis test. P-values for all categorical variables were derived from Fisher’s exact test.

b: IQR stands for inter-quartile range.

*US* ultrasound, *MRI* magnetic resonance imaging, *CT* computed tomography, *ED LOS* emergency department length of stay

**Fig. 1:**
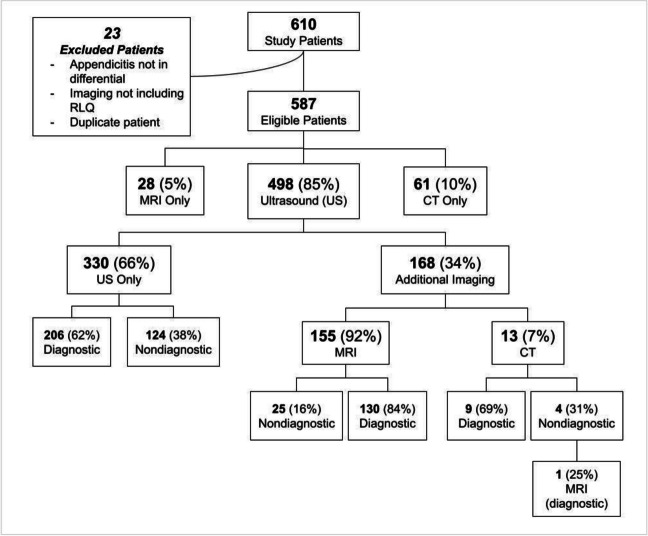
Number of patients receiving US, CT, or MRI and those receiving a combination of imaging modalities

Figure 1 demonstrates the number of patients receiving US, rapid MRI, CT or a combination of imaging modalities. 235 (47%) of the total 498 US and 169 (92%) of the total 184 rapid MRIs were diagnostic. 15 (54%) of the 28 patients who received MRI as the initial imaging modality (MRI only) were diagnosed with appendicitis. Second-line imaging (rapid MRI, CT), performed alongside US, was largely conclusive and was ordered following both diagnostic and nondiagnostic initial US findings. For patients who underwent MRI following US (155), 41 (26%) were diagnosed with appendicitis. Of the 13 additional CT scans, 100% of patients were diagnosed with appendicitis.

For patients receiving imaging at the primary care pediatric hospital, CMHH, US was diagnostic for 145 (50%) of 290 studies, while rapid MRI was diagnostic for 124 (92%) of 135 studies. For patients transferred to CMHH from another ED, US was diagnostic for 85 (43%) or 196 total studies, with rapid MRI diagnostic in 42 (93%) of 45 studies.

The sensitivity and specificity of US, rapid MRI, and CT are shown in Table 3 A, for studies that were diagnostic only (confirmed positive or negative for appendicitis) and B, combining nondiagnostic studies with negative studies. Due to the significant number of inconclusive US studies, overall (B) US sensitivity was lower than that for conclusive (A) US studies. Sensitivity and specificity for rapid MRI were high for both patients with conclusive (A) studies and overall (B) studies.

**Table 3:** Imaging Diagnosis Sensitivity & Specificity (95% CI)

	**Appendicitis**	**No Appendicitis**
**A. Diagnostic Imaging (469)**		
***Ultrasound (n = 235)***		
Positive	109	10
Negative	10	106
Sensitivity	91.6% (85.1% – 95.9%)	
Specificity	91.4% (84.7% – 95.8%)	
Positive Predictive Value	91.6% (85.1% – 95.9%)	
Negative Predictive Value	91.4% (84.7% – 95.8%)	
Prevalence	50.6% (44.1% – 57.2%)	
***MRI (n = 169)***		
Positive	56	5
Negative	0	108
Sensitivity	100.0% (93.6% – 100.0%)	
Specificity	95.6% (90.0% – 98.6%)	
Positive Predictive Value	91.8% (81.9% – 97.3%)	
Negative Predictive Value	100.0% (96.6% – 100.0%)	
Prevalence	33.1% (26.1% – 40.8%)	
***CT (n = 65)***		
Positive	60	1
Negative	0	4
Sensitivity	100.0% (94.0% – 100.0%)	
Specificity	80.0% (28.4% – 99.5%)	
Positive Predictive Value	98.4% (91.2% – 100.0%)	
Negative Predictive Value	100.0% (39.8% – 100.0%)	
Prevalence	92.3% (83.0% – 97.5%)	
**B. Overall Imaging (Diagnostic & Nondiagnostic)**		
***Ultrasound (n = 498)***		
Positive	109	10
Negative (n=116)/Nondiagnostic (n=263)	58	321
Sensitivity	65.3% (57.5% – 72.5%)	
Specificity	97.0% (94.5% – 98.5%)	
Positive Predictive Value	91.6% (85.1% – 95.9%)	
Negative Predictive Value	84.7% (80.7% – 88.2%)	
Prevalence	33.5% (29.4% – 37.9%)	
***MRI (n = 184)***		
Positive	56	5
Negative (n=108)/Nondiagnostic (n=15)	1	122
Sensitivity	98.3% (90.6% – 100.0%)	
Specificity	96.1% (91.1% – 98.7%)	
Positive Predictive Value	91.8% (81.9% – 97.3%)	
Negative Predictive Value	99.2% (95.6% – 100.0%)	
Prevalence	31.0% (24.4% – 38.2%)	
***CT (n = 74)***		
Positive	60	1
Negative (n=4)/Nondiagnostic (n=9)	7	6
Sensitivity	89.6% (79.7% – 95.7%)	
Specificity	85.7% (42.1% – 99.6%)	
Positive Predictive Value	98.4% (91.2% – 100.0%)	
Negative Predictive Value	46.2% (19.2% – 74.9%)	
Prevalence	90.5% (81.5% – 96.1%)	

*MRI* magnetic resonance imaging, *CT* computed tomography

The pathological diagnosis in patients with appendicitis in the 3 imaging modalities is shown in Table 4. Because some patients had >1 imaging study performed, there were 291 images. There were no differences between diagnostic and nondiagnostic imaging for simple or complicated appendicitis on pathology.

**Table 4:** Pathology diagnosis among patients with appendicitis

		**US**			**MRI**	
	**Diagnostic** **N = 119**	**Non-Diagnostic** **N = 48**	**P-Value** ^**a**^	**Diagnostic** **N = 56**	**Non-Diagnostic** **N = 1**	**P-Value** ^**a**^
***Diagnostic Type, n (%)***			0.77			1.00
Simple	69 (58.0)	29 (60.4)		35 (62.5)	1 (100.0)	
Complicated (Perforated)	50 (42.0) (22 Perforated)	19 (39.6) (9 Perforated)		21 (37.5) (10 Perforated)	0 (0.0) (0 Perforated)	
		**CT**				
	**Diagnostic** **N = 60**	**Non-Diagnostic** **N = 7**	**P-Value** ^**a**^			
***Diagnostic Type, n (%)***			1.00			
Simple	44 (73.3)	5 (71.4)				
Complicated (Perforated)	16 (26.7) (12 Perforated)	2 (28.6) (1 Perforated)				

a: P-value for ultrasound was derived from Pearson’s 𝜒2 test. P-values for MRI and CT were derived from Fisher’s exact test.

*US* ultrasound, *MRI* magnetic resonance imaging, *CT* computed tomography

## Discussion

Our primary finding was that US was inconclusive in more than 50% of studies performed, contributing to its poor sensitivity as the sole modality for diagnosing appendicitis. While rapid MRI demonstrated high sensitivity, it was associated with a longer LOS. This was further prolonged when combined with US.

Our reported US sensitivity and specificity are comparable to sensitivity (66.5%) and specificity (95.9%) reported at a similar institution (pediatric center nestled in a larger general hospital) [16]. This could be due to patient, provider, and system level variables that may influence the functionality of imaging in a hospital. Some patient factors may include weight and sex. As weight increases, identification of the appendix decreases [17]. In female patients, the close anatomic relationship of female reproductive organs can complicate identification of the appendix especially in the presence of ovarian cysts, torsion, or pelvic inflammatory disease [18]. Personnel factors may include less pediatric experience in sonographers. Dedicated pediatric sonographers identify the appendix at a higher rate than those without pediatric experience [16, 19]. At our and similar children’s and community hospitals, US is used less frequently in evaluation of acute appendicitis in adults. Therefore, adult sonographers and radiologists may have less pediatric specific training especially in a hospital with a lower pediatric census. This may have influenced our results. In our study, US was diagnostic in 43% of patients at community hospitals, which only improved marginally to 50% after transfer to CMHH (data not presented). Our US sensitivity was lower than that reported in a meta-analysis (89%) [20]. This reflects our real-world experience. There was a need to do further testing with another imaging modality or make a clinical decision without confirmation from the original study performed.

Our evaluation included the most recent imaging study, which was either a single image or the final US in a series of prior scans. While imaging pathways varied slightly, the ED protocol at the time recommended US as the primary imaging modality, followed by rapid MRI if nondiagnostic. CT was reserved as a secondary modality, primarily for older patients. Although an alternative diagnostic pathway for pediatric appendicitis, serial US has a lower sensitivity (79.3%) compared to combined US-MRI (98.0%) [21]. The US-MRI pathway offers a more reliable means of diagnosis in the context of inconclusive US. This improved accuracy comes with added time, possible increased cost, and patient dissatisfaction associated with longer wait times. In this analysis, we did not have cost or satisfaction data.

Use of US as the sole diagnostic imaging modality was associated with the shortest ED LOS (5.3 h), comparable to a similar study (4.98 h) [22]. With rapid MRI as the sole imaging modality, ED LOS increased by 2.6 hours, similar to Imler’s study (8.32 h) [22]. US combined with other modalities was associated with the longest ED LOS. This likely reflects inevitable delays associated with CT or rapid MRI compared to the immediate availability of US. Rapid MRI requires removal of metal objects, and MRI machines are usually in high demand, serving multiple departments for scheduled and emergency imaging. At our institution, final reads for rapid MRIs are available only during normal business hours which may add to the ED LOS. Interestingly, the US & MRI & CT pathway had a shorter LOS (5.9 hours) despite involving the greatest number of imaging modalities; however, this subgroup consisted of a single patient, thus limiting the reliability of this finding. Its shorter LOS may reflect individual patient or system factors rather than a genuine trend. Despite the slightly lower ED LOS with US and CT, US and rapid MRI may be the preferred combination to reduce the potential harms associated with radiation [23]. The decline in CT use was mirrored in our study where only 54 CT-only scans were done (9%) in pediatric patients with concern for appendicitis.

Future work will need to focus on identifying local barriers to US visualization of the appendix and decreasing rapid MRI-associated LOS. One strategy may be to implement rapid MRI-first for patients anticipated to have equivocal US (higher body-mass index, suspected ovarian pathology) to avoid delays with the combination [24]. Another could be increased availability of sonographers and radiologists with pediatric experience and extended pediatric radiologist after-hour coverage to facilitate prompt reading of MRI [25]. These may have financial implications.

## Limitations

In this retrospective study, there is a risk of incomplete data despite careful scrutiny. It is unknown if patients chose to leave or be seen at another facility, which would have impacted the false negative rate. We anticipated this to be very low because of the limited number of facilities with pediatric surgery capabilities in the area. We assumed that patients who did not have surgery and had negative imaging did not have appendicitis but cannot be certain because of limited electronic data and follow up. However, this was consistent across all modalities. We were unable to determine the rationale for the selection of imaging modality all the time which may reflect clinician experience, preference, patient demographics (e.g., body habitus, symptom severity, age of the patient), and imaging timing or availability. Thus, we cannot rule out selection bias with imaging choice. However, the ED protocol at the time called for performing an US first on all patients before an MRI and intentionally attempted to limit the use of CT in children.

## Conclusion

In our study, US did not perform as well as anticipated from the literature. These results reflect our real-world clinical experience with US for acute appendicitis in a children’s hospital within a larger general hospital. While rapid MRI had excellent sensitivity, the LOS was nearly double when added to US to diagnose appendicitis. Our findings indicate a need to improve the diagnostic capability at our center for appendicitis. Future work will need to focus on determining barriers and incorporating strategies to enhance US performance and/or decrease rapid MRI LOS.

## Supplementary Information

ESM 1 (DOCX 333 kb)

## Data Availability

The data generated and analyzed in this study are available within the paper. De-identified data was obtained from the hospital’s electronic medical record system. No external or secondary datasets were used in this analysis.
