# Supplementary material for: Diagnosis of acute appendicitis at a pediatric emergency department within a general hospital
Source: Emerg Radiol. 2026 Feb 24;33(3):503–11. doi: 10.1007/s10140-026-02442-w (PMC13233868; doi:10.1007/s10140-026-02442-w)
Supplement: Supplementary file 1 — (DOCX 333 kb) [file 10140_2026_2442_MOESM1_ESM.docx]

**Supplementary Information**

Appendix A. General Template for RLQ US


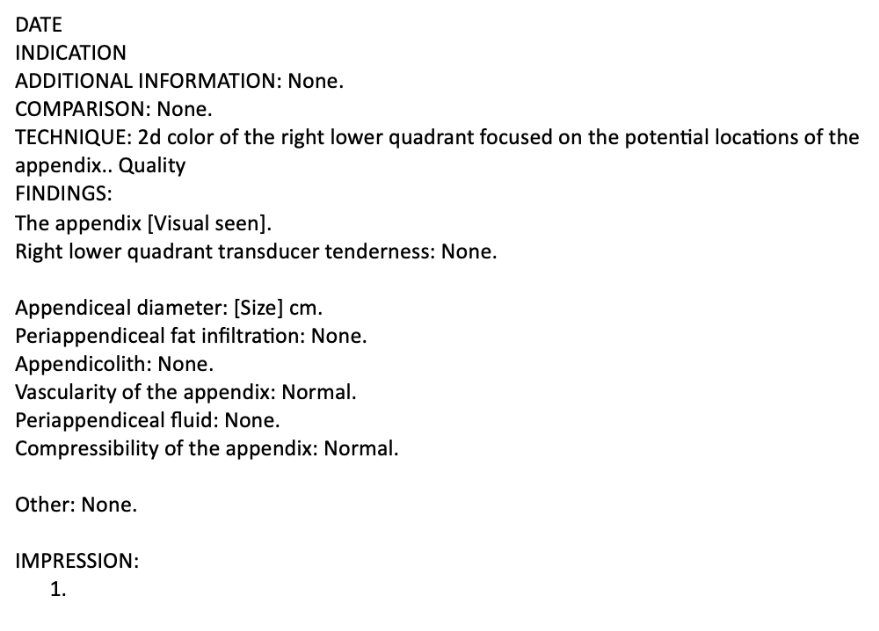


Appendix B. General Template for MR Abdomen and Pelvis Without Contrast


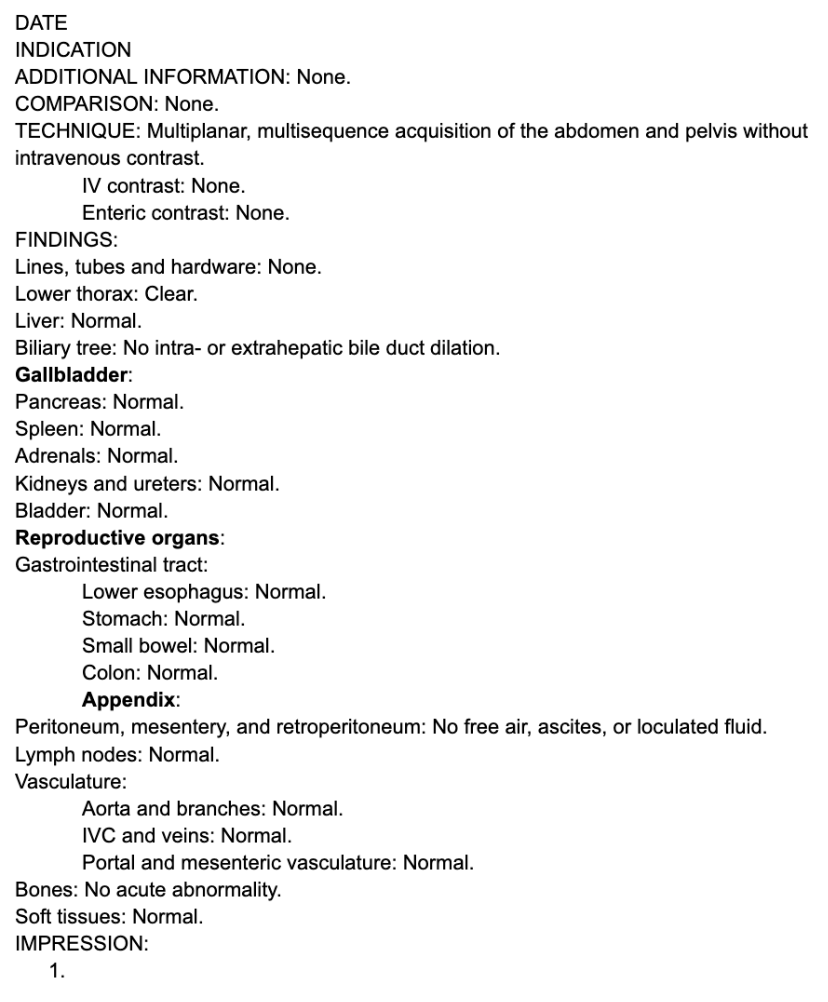


Appendix C. Rapid MRI Protocol

| SEQUENCE | PLANE |
| --- | --- |
| Single-shot T2-weighted (SSh T2) | Coronal |
| Single-shot T2-weighted fat-suppressed (SSh T2 Fat) | Coronal |
| Single-shot T2-weighted (SShot T2) | Axial |
| Single-shot T2-weighted (SShot T2) | Sagittal |
| Spin echo diffusion-weighted imaging (Spin Echo DWI) | Axial |
| Female only axial 3D | Axial |

STARD 2015 Checklist for Reporting Diagnostic Accuracy Studies:

| **Section & Topic** | **No** | **Item** | **Notes** |
| --- | --- | --- | --- |
| **TITLE OR ABSTRACT** |  |  |  |
|  | **1** | Identification as a study of diagnostic accuracy using at least one measure of accuracy  (such as sensitivity, specificity, predictive values, or AUC) | p. 1 (Abstract)  Diagnostic accuracy study measuring sensitivity and specificity |
| **ABSTRACT** |  |  |  |
|  | **2** | Structured summary of study design, methods, results, and conclusions  (for specific guidance, see STARD for Abstracts) | p. 1 |
| **INTRODUCTION** |  |  |  |
|  | **3** | Scientific and clinical background, including the intended use and clinical role of the index test | p. 2 |
|  | **4** | Study objectives and hypotheses | p. 2 |
| **METHODS** |  |  |  |
| *Study design* | **5** | Whether data collection was planned before the index test and reference standard were performed (prospective study) or after (retrospective study) | p. 2  Retrospective |
| *Participants* | **6** | Eligibility criteria | p. 3 |
|  | **7** | On what basis potentially eligible participants were identified  (such as symptoms, results from previous tests, inclusion in registry) | p. 3 |
|  | **8** | Where and when potentially eligible participants were identified (setting, location, and dates) | p. 2-3  CMHH, 01/01/2019 - 06/30/2019 |
|  | **9** | Whether participants formed a consecutive, random or convenience series | p. 3  Consecutive |
| *Test methods* | **10a** | Index test, in sufficient detail to allow replication | p. 3  Ultrasound |
|  | **10b** | Reference standard, in sufficient detail to allow replication | p. 3  Rapid Magnetic Resonance Imaging |
|  | **11** | Rationale for choosing the reference standard (if alternatives exist) | n/a |
|  | **12a** | Definition of and rationale for test positivity cut-offs or result categories  of the index test, distinguishing pre-specified from exploratory | p. 3-4  Definition of diagnostic vs nondiagnostic imaging |
|  | **12b** | Definition of and rationale for test positivity cut-offs or result categories  of the reference standard, distinguishing pre-specified from exploratory | p. 3-4 |
|  | **13a** | Whether clinical information and reference standard results were available  to the performers/readers of the index test | p. 3  One blinded member reviewed EMR and data, all imaging reads blinded due to retrospective nature of study |
|  | **13b** | Whether clinical information and index test results were available  to the assessors of the reference standard | p. 3 |
| *Analysis* | **14** | Methods for estimating or comparing measures of diagnostic accuracy | p. 4  Sensitivity, Specificity calculated for diagnostic (unequivocally positive or negative) studies and for diagnostic + nondiagnostic (equivocal or when appendix not visualized) |
|  | **15** | How indeterminate index test or reference standard results were handled | p. 4  Further imaging/combination of imaging modalities were obtained |
|  | **16** | How missing data on the index test and reference standard were handled | p. 3  Exclusion criteria |
|  | **17** | Any analyses of variability in diagnostic accuracy, distinguishing pre-specified from exploratory | p. 4  By imaging modality or combination utilized |
|  | **18** | Intended sample size and how it was determined | p. 5  Based on all consecutive eligible patients in the study period |
| **RESULTS** |  |  |  |
| *Participants* | **19** | Flow of participants, using a diagram | p. 6 (Figure 1) |
|  | **20** | Baseline demographic and clinical characteristics of participants | p. 5 (Tables 1-2) |
|  | **21a** | Distribution of severity of disease in those with the target condition | p. 6  (Appendicitis/Positive/Pathology) |
|  | **21b** | Distribution of alternative diagnoses in those without the target condition | p. 5  No Appendicitis/Negative) |
|  | **22** | Time interval and any clinical interventions between index test and reference standard | p. 5  n/a, Length of Stay (LOS) |
| *Test results* | **23** | Cross tabulation of the index test results (or their distribution)  by the results of the reference standard | p. 6, 15 (Table 3) |
|  | **24** | Estimates of diagnostic accuracy and their precision (such as 95% confidence intervals) | p. 6  Sensitivity and Specificity with 95% confidence intervals |
|  | **25** | Any adverse events from performing the index test or the reference standard | None reported. |
| **DISCUSSION** |  |  |  |
|  | **26** | Study limitations, including sources of potential bias, statistical uncertainty, and generalizability | p. 8 |
|  | **27** | Implications for practice, including the intended use and clinical role of the index test | p. 7-8 |
| **OTHER INFORMATION** |  |  |  |
|  | **28** | Registration number and name of registry | n/a |
|  | **29** | Where the full study protocol can be accessed | Study methods, analyses, and protocols are described in the manuscript; no separate protocol was published. |
|  | **30** | Sources of funding and other support; role of funders | n/a |
